# Autochthonous Ratborne Seoul Virus Infection in Woman with Acute Kidney Injury

**DOI:** 10.3201/eid2612.200708

**Published:** 2020-12

**Authors:** Jörg Hofmann, Elisa Heuser, Sabrina Weiss, Beate Tenner, Konrad Schoppmeyer, Jutta Esser, Christiane Klier, Stephan Drewes, Rainer G. Ulrich, Detlev H. Kruger

**Affiliations:** Charité–Universitätsmedizin Berlin, Berlin, Germany (J. Hofmann, S. Weiss, B. Tenner, D.H. Kruger);; Friedrich-Loeffler-Institut, Greifswald-Insel Riems, Germany (E. Heuser, S. Drewes, R.G. Ulrich);; Euregio-Klinik, Medizinische Klinik II, Nordhorn, Germany (K. Schoppmeyer);; Laborarztpraxis Osnabrück, Georgsmarienhütte, Germany (J. Esser);; German Center for Infection Research, Partner Site Hamburg-Lübeck-Borstel-Insel Riems, Germany (E. Heuser, R.G. Ulrich);; Public Health Agency of Lower Saxony, Hannover, Germany (C. Klier)

**Keywords:** Seoul virus, hantavirus, renal failure, respiratory distress, zoonoses, hantavirus pulmonary syndrome, Germany, viruses, rats

## Abstract

Outside Asia, Seoul virus (SEOV) is an underestimated pathogen. In Germany, autochthonous SEOV-associated hantavirus disease has not been unequivocally diagnosed. We found clinical and molecular evidence for SEOV infection in a young woman; her pet rat was the source of infection.

Hantavirus infections cause febrile and often life-threatening zoonoses known as hemorrhagic fever with renal syndrome and hantavirus cardiopulmonary syndrome. Human pathogenic hantavirus species usually are carried by specific rodent reservoirs, which shed infectious virus in their excreta (*1*).

Seoul virus (SEOV), a species within the genus *Orthohantavirus*, is hosted by Norway or brown rats (*Rattus norvegicus*) and other *Rattus* species as main reservoir. SEOV-associated hantavirus disease is characterized by fever, acute kidney injury, often hepatitis and gastroenteritis, associated with transient thrombocytopenia and proteinuria (*2*,*3*). Most clinical cases are known to originate from China and South Korea; however, SEOV infection can occur worldwide because of the global distribution of Norway rats in the wild. Moreover, human infection has been described from contact with breeder rats (laboratory rats and laboratory rat–derived tissue cultures), pet rats, and feeder rats (*3*–*6*).

SEOV-caused hantavirus disease, especially in areas outside Asia to which it is not endemic, is often misdiagnosed, perhaps because of its sometimes mild/atypical clinical presentation and healthcare providers’ low clinical awareness (*2*,*3*). A lack of appropriate routine diagnostic tools also complicate the correct diagnosis. SEOV nucleocapsid protein shares a high antigenic similarity to related orthohantaviruses, such as Hantaan virus (HTNV) and Dobrava-Belgrade virus (DOBV), and is not always included in commercial assays (*1*,*7*).

Therefore, the use of molecular methods is the best way to unequivocally prove SEOV infections in Europe. Molecular evidence for SEOV infection has been found in patients from France and the Netherlands (*6*,*8*). Molecularly proven SEOV hantavirus disease in a German patient was reported in 2018, but the infection probably was acquired in Indonesia (*7*). Except for this travel-associated infection, neither SEOV-specific antibodies nor SEOV RNA had been detected in humans in Germany.

In October 2019, an 18-year-old woman was admitted to the intensive care unit of a hospital in Nordhorn in northwestern Germany with high fever and in critical condition. During the clinical course of her illness, acute kidney injury, gastroenteritis, and hepatopathy developed. Thrombocytes were lowest at day 3 and normal from day 6 on. Leukocytosis was evident during days 6–8, C-reactive protein as an inflammation parameter was above normal, peaked on day 2, and then decreased continuously until day 12. Serum creatinine and urea were elevated, and glomerular filtration rate was reduced with most critical values of all 3 parameters on day 8. We also detected proteinuria. The >3-fold increase in serum creatinine concentration from day 1 to day 8 is consistent with an acute kidney injury severity level 3 in the 3-stage KDIGO (Kidney Disease: Improving Global Outcomes) classification (*9*). These parameters of kidney function reached normal or nearly normal levels on day 12. Liver enzymes were elevated during the entire period and peaked on day 3 (Table). After receiving antimicrobial treatment and treatment for her symptoms, the patient was discharged from the hospital on day 13 in largely normal condition.

**Table:** Biochemical parameters of the patient with Seoul virus during hospitalization, Germany, 2018*

Parameter (reference range)	Day 1	Day 2	Day 3	Day 4	Day 6	Day 7	Day 8	Day 9	Day 11	Day 12
Platelets (150–400/μL)	183	93	73	89	167	185	334	379	515	537
Leukocytes (4–10/μL)	3.4	4.7	4.4	5.5	10.4	10.8	10.1	9.1	9.4	8.6
CRP (0–0.5 mg/dL)	4.7	12.4	11.5	7.8	6.2	4.8	4.1	3.6	1.4	0.9
Serum creatinine (0.5–0.9 mg/dL)	0.92	1.42	1.93	1.81	2.27	2.72	2.93	2.33	1.18	1.16
Serum urea (16.6–48.5 mg/dL)	ND	41.5	ND	55.2	63.3	67.5	67.8	53.0	17.9	17.0
GFR (>89 mL/min)	91	54	37	40	31	25	22	30	67	69
Protein in urine (0 mg/dL)	ND	ND	75	ND	ND	75	75	ND	ND	–
γGT (6–42 U/L)	67	202	206	172	187	177	159	141	110	156
ALT (10–35 U/L)	28	164	233	140	117	96	67	55	39	54

*ALT, alanine aminotransferase; CRP, C-reactive protein; γGT, gamma-glutamyltransferase; GFR, glomerular filtration rate; –, negative; ND, not determined.

Serologic diagnostic approaches were based on *recom*Line HantaPlus IgG and IgM immunoblot assays (Mikrogen GmbH, https://www.mikrogen.de). The *recom*Line IgM blot showed strong reactivity to DOBV, HTNV, and SEOV nucleocapsid antigens, and in the IgG blot, we found a single weak reactivity to HTNV. A follow-up sample drawn 2 months after discharge revealed comparable band intensities in the IgM blot. The IgG blot showed a strong HTNV band but no DOBV or SEOV reactivities. However, neither DOBV nor HTNV are prevalent in the patient’s residential area, and she reported not traveling.

We conducted molecular virus typing. A serum sample collected on day 5 of hospitalization was tested by the pan-hanta reverse transcription PCR (RT-PCR) addressing a 412-nt region of the viral large (L) segment (*10*). The identified nucleotide sequence demonstrated SEOV infection.

The patient reported that she kept Norway rats as pets in her flat. RT-PCR investigation of lung tissue of 1 of these rats yielded an L segment sequence identical to the patient-derived sequence (Figure). A subsequent small (S) segment RT-PCR enabled amplification of a 673-nt sequence from both the patient and the pet rat. Sequence alignment showed only a single silent nucleotide exchange. The analyzed S segment sequences exhibited the highest similarity to breeder-rat derived SEOV strains from the Netherlands and United Kingdom (Figure). The identities of the patient- and pet rat-derived sequences support the zoonotic transmission of the virus to the woman.

**Figure:**
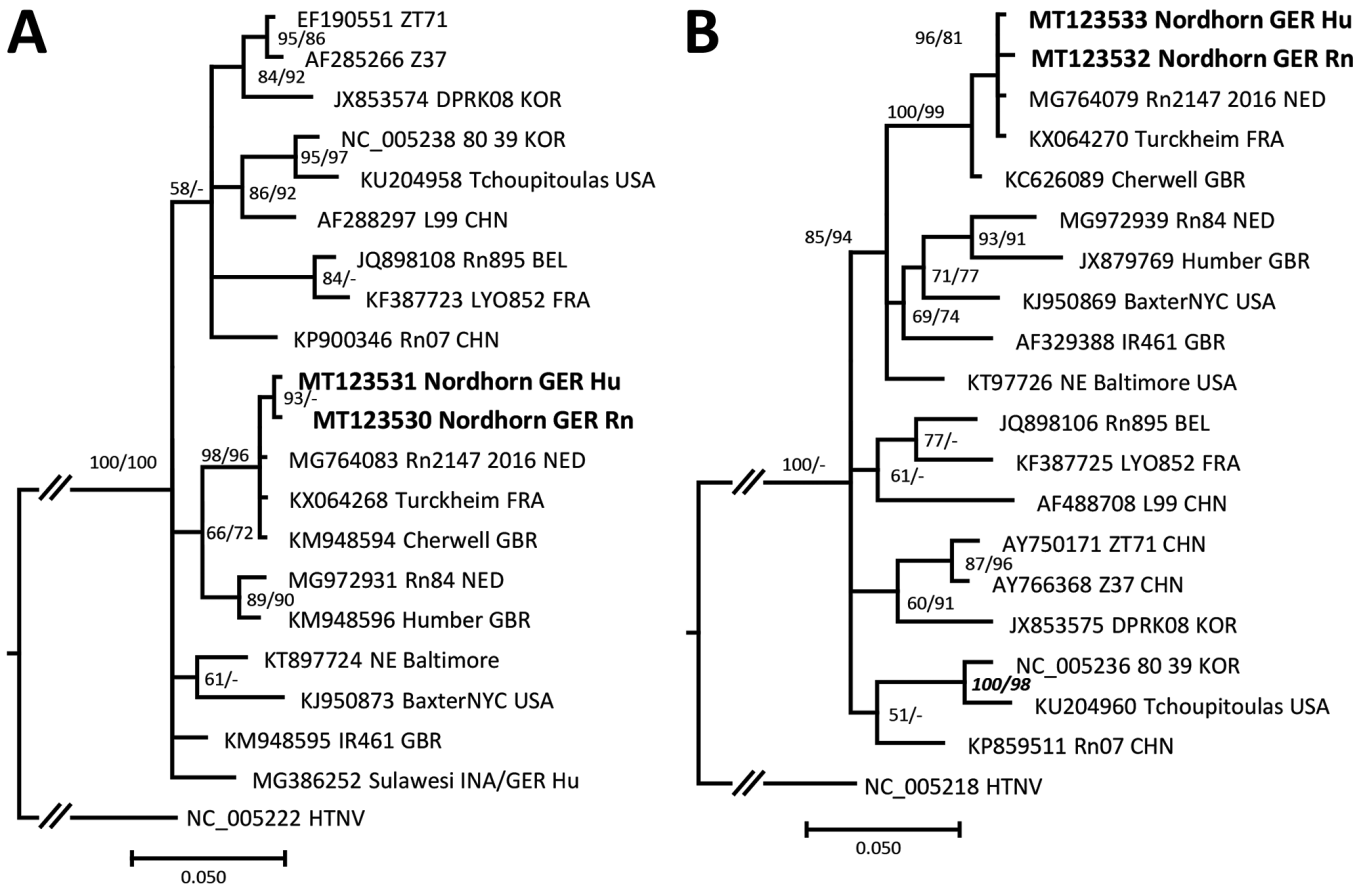
Molecular phylogenetic analysis of the amplified large (L) and small (S) segment regions of human and rat origin from Nordhorn/Germany (strains Nordhorn GER Hu and Nordhorn GER Rn, designated in bold). The consensus tree is based on a 412-nt region of the L segment (A) and a 673-nt region of the S segment (B). Alignments were constructed with Bioedit software package version7.2.5) (https://bioedit.software.informer.com) using the Clustal W Multiple Alignment algorithm. The best fitting substitution model was determined with jModeltest version 2.1.10 (https://github.com/ddarriba/jmodeltest2). Trees were reconstructed with MrBayes version 3.2.6 (http://www.mrbayes.net) and FasttreeMP version 2.1.10 (http://microbesonline.org/fasttree) executed on the CIPRES portal (https://www.phylo.org) according to maximum-likelihood and Markov chain Monte Carlo algorithms. The consensus tree is based on Bayesian analyses with 2 × 10^6^ generations, a burn-in phase of 25%, and the Hasegawa-Kishono-Yano substitution model with gamma distribution. Bootstrap values were transferred to the Bayesian tree behind posterior probabilities only if they were >50% and if branches of both trees were consistent. Hantaan virus was used as outgroup. The L and S segment sequences were deposited in GenBank under accession nos. MT123530–33. At the end of the strain names the country of origin is given: BEL, Belgium; CHN, China; FRA, France; GBR, Great Britain; GER, Germany; INA, Indonesia; KOR, South Korea; NED, the Netherlands; USA, United States. Scale bars indicate nucleotide substitutions per site.

This case illustrates the importance of clinical awareness for SEOV infection after contact with rats. Along with this human case, we report a molecularly proven SEOV infection in a pet rat in Germany. More information regarding the SEOV prevalence in domestic and wild rat populations in Germany is needed to assess the risk for infection in the general public, pet rat owners, and breeder-rat handlers.
